# Investigating Spectrum of Biological Activity of 4- and 5-Chloro-2-hydroxy-*N*-[2-(arylamino)-1-alkyl-2-oxoethyl]benzamides

**DOI:** 10.3390/molecules16032414

**Published:** 2011-03-14

**Authors:** Ales Imramovsky, Matus Pesko, Katarina Kralova, Marcela Vejsova, Jirina Stolarikova, Jarmila Vinsova, Josef Jampilek

**Affiliations:** 1 Institute of Organic Chemistry and Technology, Faculty of Chemical Technology, University of Pardubice, Studentska 573, 532 10 Pardubice, Czech Republic; 2 Institute of Chemistry, Faculty of Natural Sciences, Comenius University, Mlynska dolina Ch-2, 842 15 Bratislava, Slovakia; 3 Department of Clinical Microbiology, Charles University Medical School and Teaching Hospital, Sokolska 581, Hradec Kralove 500 05, Czech Republic; 4 Laboratory for Mycobacterial Diagnostics and TB, Institute of Public Health in Ostrava, Partyzanske namesti 7, 702 00 Ostrava, Czech Republic; 5 Department of Inorganic and Organic Chemistry, Faculty of Pharmacy in Hradec Kralove, Charles University in Prague, Heyrovskeho 1203, 500 05 Hradec Kralove, Czech Republic; 6 Department of Chemical Drugs, Faculty of Pharmacy, University of Veterinary and Pharmaceutical Sciences, Palackeho 1/3, 612 42 Brno, Czech Republic

**Keywords:** salicylanilide derivates, lipophilicity, *in vitro* antimycobacterial activity, *in vitro* antifungal activity, *in vitro* antibacterial activity, PET inhibition, spinach chloroplasts, structure–activity relationships

## Abstract

In this study, a series of twenty-two 5-chloro-2-hydroxy-*N*-[2-(arylamino)-1-alkyl-2-oxoethyl]benzamides and ten 4-chloro-2-hydroxy-*N*-[2-(arylamino)-1-alkyl-2-oxoethyl]benzamides is described. The compounds were analyzed using RP-HPLC to determine lipophilicity. Primary *in vitro* screening of the synthesized compounds was performed against mycobacterial, bacterial and fungal strains. They were also evaluated for their activity related to the inhibition of photosynthetic electron transport (PET) in spinach (*Spinacia oleracea* L.) chloroplasts. The compounds showed biological activity comparable with or higher than the standards isoniazid, fluconazole, penicillin G or ciprofloxacin. For all the compounds, the relationships between the lipophilicity and the chemical structure of the studied compounds as well as their structure-activity relationships are discussed.

## 1. Introduction

Salicylanilides (2-hydroxy-*N*-phenylbenzamides) are an important class of aromatic compounds with a wide range of pharmacological activities. A number of them show antibacterial [1,2,3], antimycobacterial [4,5,6] and antifungal [4,7] as well as anti-inflammatory [8] or antineoplastic activities [9,10,11]. In spite of their promise as potential drugs, certain physico-chemical properties of salicylanilides, for example low solubility, prevented their widespread use in clinical practice. Thus, improvement of the physical-chemical properties of salicylanilides is an interesting and vital area of research. 

**Scheme 1:**
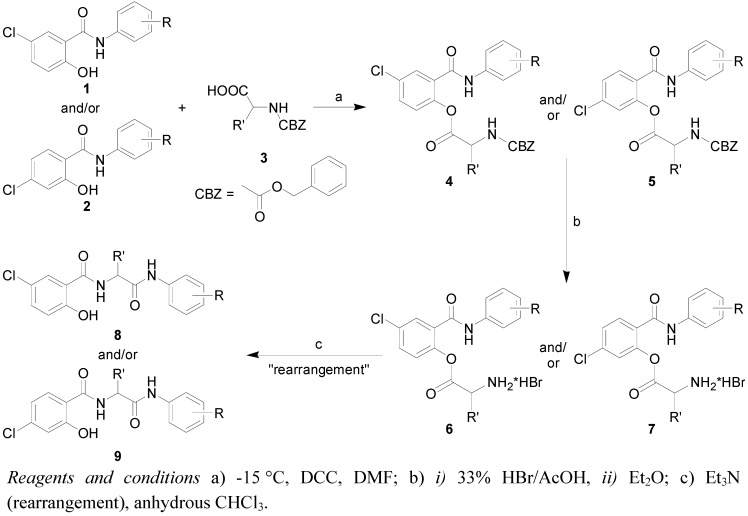
Synthetic pathway for preparation of target substituted 5-chloro-2-hydroxy-*N*-[2-(arylamino)-1-alkyl-2-oxoethyl]benzamides **8a**-**8v** and 4-chloro-2-hydroxy-*N*-[2-(aryl-amino)-1-alkyl-2-oxoethyl]benzamides **9a**-**9j**.

We have focused our efforts on design and synthesis of original *O*-substituted derivates of salicylanilides. Promising results of antimycobacterial evaluation of some salicylanilides acetates [4] inspired us to design more sophisticated derivates of salicylanilides – amino acid esters. The design strategy of these potential antimicrobial active agents was mainly oriented towards the synthesis of a prodrug form of most antitubercular active salicylanilides by attaching an amino acid. The phenolic hydroxyl group of the most antitubercular active salicylanilides was esterified by several *N*-benzyloxy-carbonyl α-amino acids. More lipophilic amino acids such as glycine, (*R* and *S*)-alanine, (*R* and *S*)-valine and (*R* and *S*)-phenylalanine were chosen. The prepared *N*-protected esters were tested for their antifungal and antimycobacterial activity; promising results were published recently [5,6,7]. Subsequent processing using standard reactions yielded unexpected products (4-chloro- and 5-chloro-*N*-salicylamides for detailed structure see Scheme 1) that were described, including proposed reaction mechanism, and published [12,13,14].

It was expected that chloro-2-hydroxy-*N*-(arylalkyl)benzamides, compounds with a special skeleton originating from biologically active salicylanilides, would have interesting biological properties. A series of these compounds was prepared, and most of them were tested for their antimycobacterial, antifungal and antibacterial activity. As the presence of an amide (-NHCO-) group in acylanilides, phenylcarbamates, ureas, *etc.*, is characteristic of a number of herbicides acting as photosynthesis inhibitors [15,16,17,18,19,20,21,22,23,24,25], the compounds were also evaluated for their photosynthesis-inhibiting activity (the inhibition of photosynthetic electron transport) in spinach chloroplasts (*Spinacia oleracea* L.). These compounds bind reversibly to photosystem II (PS II), a membrane-protein complex in the thylakoid membranes, which catalyses oxidation of water and reduction of plastoquinone [15], and thereby inhibit photosynthesis [26,27,28,29,30]. For example, in the presence of R' substituted salicylanilides the decreased intensity of the fluorescence emission band at 686 nm (belonging to the chlorophyll-protein complexes mainly in PS II [31]) suggested PS II as the site of action of the studied inhibitors [24].

Many low molecular weight drugs cross biological membranes through passive transport, which strongly depends on their lipophilicity, therefore this physico-chemical parameter of the prepared compounds was determined by means of the RP-HPLC. Relationships between the structure and *in vitro* antimicrobial activities or/and inhibitory activity related to inhibition of photosynthetic electron transport (PET) in spinach chloroplasts of the new compounds are discussed.

## 2. Results and Discussion

### 2.1. Chemistry

Preparation of substituted 5-chloro-2-hydroxy-*N*-[2-(arylamino)-1-alkyl-2-oxoethyl]benzamides **8a**-**v** and 4-chloro-2-hydroxy-*N*-[2-(arylamino)-1-alkyl-2-oxoethyl]benzamides **9a**-**j** was carried out by two-step synthesis with a subsequent rearrangement (Scheme 1). The first step was dicyclocarbodiimide (DCC) mediated esterification of *N*-protected amino acids **3** (benzyloxycarbonyl group as protection) with selected antimicrobial active salicylanilides **1** and **2** in dry dimethylformamide (DMF). Esters **4** and **5** were obtained optically pure in high yields [5,7].

Acidolysis (33% HBr in acetic acid) was used as a method for *N*-deprotection and this method gave appropriate hydrobromide salts **6** and **7** in almost quantitative yields. Subsequent amino group liberation by triethylamine in anhydrous chloroform yielded unexpected rearranged products **8** and **9** [12,13]. This methodology is general and is not dependent on the substitution of the aromatic salicylanilide ring as well as on the amino acid type. A small combinatorial library of discussed compounds **8** and **9** was recently synthesized and published [22]. The compounds could be divided into six groups based on their chemical structure: Group 1 includes 5-Cl/3-Cl substituted diamides **8a**-**8g**; Group 2 contains 5-Cl/4-Cl derivatives **8h**-**8l**; Group 3 includes 5-Cl/3,4-Cl derivatives **8m**-**8r**; Group 4 includes 5-Cl/4-Br derivatives **8s**-**8v**; Group 5 contains 4-Cl/4-Cl derivatives **9a**-**9f**; and Group 6 is composed of 4-Cl/4-X diamides **9g**-**9j**.

### 2.2. Lipophilicity

Lipophilicity is a property that has a major effect on absorption, distribution, metabolism, excretion and toxicity properties as well as pharmacological activity, because drugs cross biological membranes through passive transport, which strongly depends on their lipophilicity. Lipophilicity has been studied and applied as an important drug property for decades [32].

**Figure 1:**
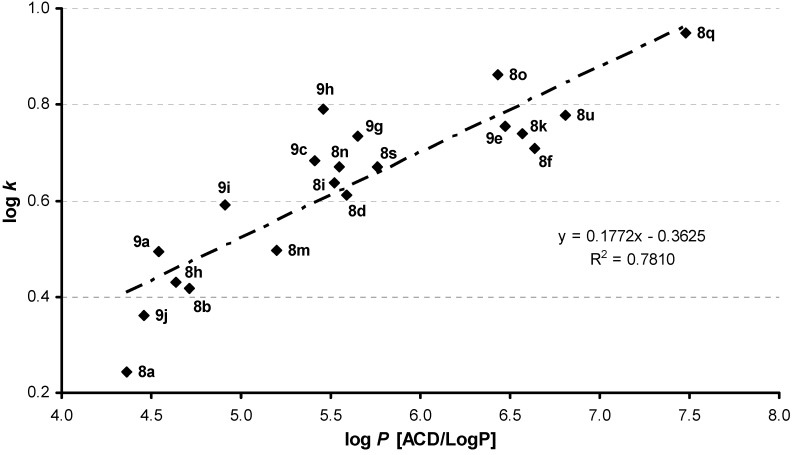
Relationship between calculated log *P* data (ACD/LogP) and experimentally found log *k* values. If both enantiomers were prepared, only (*S*)-enantiomers are illustrated due to similar log *k* values.

Hydrophobicities (log *P*/Clog *P*) of compounds **8** and **9** were calculated using two commercially available programs (ChemOffice Ultra 10.0 and ACD/LogP) and measured by means of RP-HPLC determination of capacity factors *k* with subsequent calculation of log *k*. The procedure was performed under isocratic conditions with methanol as an organic modifier in the mobile phase using an end-capped non-polar C_18_ stationary RP column. The results are shown in Table 1 and illustrated in Figure 1.

Both used programs did not resolve lipophilicity parameters within the series of enantiomers, as expected. The program ChemOffice did not resolve various lipophilicity values of individual positional isomers, in particular, the same log *P*/Clog *P* data were calculated for compounds **8a**-**8g** from Group 1, **8h**-**8l** from Group 2, **9h**-**9f** from Group 5, **8s**-**8v** from Group 4 and compound 4-Cl/4-Br (**9g**) from Group 6.

The results obtained with all the compounds show that the experimentally-determined lipophilicities (log *k*) of the discussed compounds are in accordance with the calculated values of compounds **8** and **9** as shown in Figure 1. Compounds series **9** substituted by chlorine in C_(4)_ position showed higher lipophilicity than compounds series **8** with C_(5)_ chlorine substitution, although program ACD/LogP showed an opposite trend. According to the program ACD/LogP, compounds **5a**-**5g** substituted by chlorine in C'_(3)_ position showed higher lipophilicity than compounds **5h**-**5l** with C'_(4)_ chlorine substitution, but experimental log *k* for C'_(4)_ chlorine substitution is higher. As expected, the lipophilicity of the compounds substituted in the anilide part of the molecule increases as follows: OCH_3_ < CH_3_ < Cl < Br < CF_3_ < di-Cl, although program ACD/LogP calculates higher log *P* for Br derivative **9g** than CF_3_ substituted compound **9i**. The lipophilicity factor log *k* as well as calculated log *P* (ACD/LogP) and log *P*/Clog *P* values (ChemOffice) increase, depending on amino acid type (glycine, alanine, valine and phenylalanine), that is, the lipophilicity of the compounds substituted in R^3^ increases as follows: H < CH_3_ < CH(CH_3_)_2_ < CH_2_C_6_H_5_.

Log *k* data specify lipophilicity within this series of the discussed compounds, and all the chiral compounds were measured several times with the same results. The determined differences in log *k* parameters for individual *R*/*S*-enantiomers cannot be explained on the basis of the results presented here.

**Table 1:** Comparison of calculated lipophilicities (log *P*/Clog *P*) with determined log *k* values, Hammett's parameter (σ) and bulk parameter (MR, reflecting bulkiness).

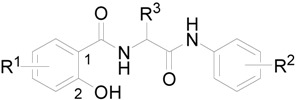											
Comp.		R^1^	R^2^	R^3^	log *k*		log *P*/Clog *P* ChemOffice	log *P* ACD/LogP	σ_R2_[33]	MR_R3_[33]	
**GROUP 1**	**8a**	5-Cl	3-Cl	H		0.2439	2.42 / 4.33156	4.36 ± 0.47	0.373	0	
	**8b**	5-Cl	3-Cl	(*S*)-CH_3_		0.4166	2.91 / 4.64056	4.71 ± 0.48	0.373	4.7	
	**8c**	5-Cl	3-Cl	(*R*)-CH_3_		0.4153	2.91 / 4.64056	4.71 ± 0.48	0.373	4.7	
	**8d**	5-Cl	3-Cl	(*S*)-CH(CH_3_)_2_		0.6124	3.80 / 5.56856	5.59 ± 0.48	0.373	14.0	
	**8e**	5-Cl	3-Cl	(*R*)-CH(CH_3_)_2_		0.6114	3.80 / 5.56856	5.59 ± 0.48	0.373	14.0	
	**8f**	5-Cl	3-Cl	(*S*)-CH_2_C_6_H_5_		0.7098	4.58 / 6.05856	6.64 ± 0.49	0.373	29.0	
	**8g**	5-Cl	3-Cl	(*R*)-CH_2_C_6_H_5_		0.7066	4.58 / 6.05856	6.64 ± 0.49	0.373	29.0	
**GROUP 2**	**8h**	5-Cl	4-Cl	(*R*)-CH_3_		0.4308	2.91 / 4.64056	4.64 ± 0.47	0.227	4.7	
	**8i**	5-Cl	4-Cl	(*S*)-CH(CH_3_)_2_		0.6371	3.80 / 5.56856	5.52 ± 0.48	0.227	14.0	
	**8j**	5-Cl	4-Cl	(*R*)-CH(CH_3_)_2_		0.6125	3.80 / 5.56856	5.52 ± 0.48	0.227	14.0	
	**8k**	5-Cl	4-Cl	(*S*)-CH_2_C_6_H_5_		0.7394	4.58 / 6.05856	6.57 ± 0.49	0.227	29.0	
	**8l**	5-Cl	4-Cl	(*R*)-CH_2_C_6_H_5_		0.7329	4.58 / 6.05856	6.57 ± 0.49	0.227	29.0	
**GROUP 3**	**8m**	5-Cl	3,4-Cl	H		0.4971	2.98 / 5.01472	5.20 ± 0.49	0.600	0	
	**8n**	5-Cl	3,4-Cl	(*S*)-CH_3_		0.6698	3.47 / 5.32372	5.55 ± 0.50	0.600	4.7	
	**8o**	5-Cl	3,4-Cl	(*S*)-CH(CH_3_)_2_		0.8623	4.35 / 6.25172	6.43 ± 0.50	0.600	14.0	
	**8p**	5-Cl	3,4-Cl	(*R*)-CH(CH_3_)_2_		0.8614	4.35 / 6.25172	6.43 ± 0.50	0.600	14.0	
	**8q**	5-Cl	3,4-Cl	(*S*)-CH_2_C_6_H_5_		0.9485	5.14 / 6.74172	7.48 ± 0.51	0.600	29.0	
	**8r**	5-Cl	3,4-Cl	(*R*)-CH_2_C_6_H_5_		0.9408	5.14 / 6.74172	7.48 ± 0.51	0.600	29.0	
**GROUP 4**	**8s**	5-Cl	4-Br	(*S*)-CH(CH_3_)_2_		0.6709	4.07 / 5.71856	5.76 ± 0.54	0.232	14.0	
	**8t**	5-Cl	4-Br	(*R*)-CH(CH_3_)_2_		0.6646	4.07 / 5.71856	5.76 ± 0.54	0.232	14.0	
	**8u**	5-Cl	4-Br	(*S*)-CH_2_C_6_H_5_		0.7779	4.86 / 6.20856	6.81 ± 0.55	0.232	29.0	
	**8v**	5-Cl	4-Br	(*R*)-CH_2_C_6_H_5_		0.7673	4.86 / 6.20856	6.81 ± 0.55	0.232	29.0	
**GROUP 5**	**9a**	4-Cl	4-Cl	(*S*)-CH_3_		0.4934	2.91 / 4.64056	4.54 ± 0.47	0.227	4.7	
	**9b**	4-Cl	4-Cl	(*R*)-CH_3_		0.4921	2.91 / 4.64056	4.54 ± 0.47	0.227	4.7	
	**9c**	4-Cl	4-Cl	(*S*)-CH(CH_3_)_2_		0.6839	3.80 / 5.56856	5.41 ± 0.48	0.227	14.0	
	**9d**	4-Cl	4-Cl	(*R*)-CH(CH_3_)_2_		0.6832	3.80 / 5.56856	5.41 ± 0.48	0.227	14.0	
	**9e**	4-Cl	4-Cl	(*S*)-CH_2_C_6_H_5_		0.7540	4.58 / 6.05856	6.47 ± 0.49	0.227	29.0	
	**9f**	4-Cl	4-Cl	(*R*)-CH_2_C_6_H_5_		0.7479	4.58 / 6.05856	6.47 ± 0.49	0.227	29.0	
**GROUP 6**	**9g**	4-Cl	4-Br	(R)-CH(CH3)2		0.7353	4.07 / 5.71856	5.65 ± 0.54	0.232	14.0	
	**9h**	4-Cl	4-CF3	(S)-CH(CH3)2		0.7892	4.16 / 5.93176	5.46 ± 0.51	0.740	14.0	
	**9i**	4-Cl	4-CH3	(S)-CH(CH3)2		0.5902	3.72 / 5.09696	4.91 ± 0.46	-0.170	14.0	
	**9j**	4-Cl	4-OCH3	(S)-CH(CH3)2		0.3623	3.11 / 4.67336	4.46 ± 0.48	-0.270	14.0	

### 2.3. Biological activities

The compounds showed a wide range of biological activities, and some noteworthy structure-activity relationships were observed. All the results are shown in Table 2. Generally, all the discussed compounds (especially compounds **8b**, **8q**, **8r**) exhibited lower solubility in comparison with the initial esters [5,7].

#### 2.3.1. Antimycobacterial screening

The discussed amides were tested against three *Mycobacteria* strains. According to the results of *in vitro* evaluation (see Table 2), all compounds demonstrated noteworthy effects. Activities of some benzamides, especially against *M. avium* and *M. kansasii*, considerably exceeded the activity of isoniazid (INH) used as an internal standard. The discussed compounds can be divided according to the activities against individual *Mycobacteria* strains, and structure-activity relationships within Group 1, Group 2 and Group 4 can be discussed. Compound **8b** showed the lowest activity due to precipitation in the testing medium, contrary to its (*R*)-enantiomer **8c**. Also compounds **8q** and **8r** precipitated from testing medium, therefore **8b**, **8q** and **8r** were excluded from the below SAR discussion.

It can be concluded that compound *N*-{(1*R*)-1-benzyl-2-[(4-chlorophenyl)amino]-2-oxoethyl}-5-chloro-2-hydroxybenzamide (**8l**) and *N*-{(1*R*)-1-benzyl-2-[(4-chlorophenyl)amino]-2-oxoethyl}-4-chloro-2-hydroxybenzamide (**9f**) showed the highest antitubercular/antimycobacterial activity, see Table 2.

**Table 2:** Antimycobacterial activity (MIC/IC_90_) of compounds in comparison with isoniazid (INH) standard; *in vitro* antifungal and antibacterial activity (MIC/IC_50_ or IC_90_) of compounds compared with fluconazole (FLU), penicillin G (PEN) and ciprofloxacin (CPF) standards; and IC_50_ values of discussed compounds related to photosynthetic electron transport (PET) inhibition in spinach chloroplasts in comparison with 3-(3,4-dichlorophenyl)-1,1-dimethylurea (DCMU) standard. (MIC-minimum inhibitory concentration, ND-not determined, MTB-*Mycobacterium tuberculosis*, MA-*M. avium*, MK-*M.kansasii*, CT-*Candida tropicalis*, CK-*C. krusei*, TM-*Trichophyton mentagrophytes*, SA-*S. aureus*, MRSA-methicilin resistant *Staphylococcus aureus*, SE-*S. epidermidis*, EF-*Enterococcus sp.*)

Comp.		MIC [µmol/L]										PET IC_50_ [μmol/L]
		MTB *^a^*	MA *^a^*	MK *^a^*	CT *^b^*	CK *^b^*	TM *^c^*	SA *^d^*	MRSA *^d^*	SE *^d^*	EF *^d^*	
		*14h*	*14h*	*14h*	*24h*	*24h*	*72h*	*24h*	*24h*	*24h*	*24h*	
		*21h*	*21h*	*21h*	*48h*	*48h*	*120h*	*48h*	*48h*	*48h*	*48h*	
**GROUP 1**	**8a**	125	62.5	250	125	125	125	>250	>250	>250	>250	378.6
		125	62.5	250	125	125	125	>250	>250	>250	>250	
	**8b*^e^***	250	500	500	>125	>125	125	>250	>250	>250	>250	41.7
		>500	>500	>500	>125	>125	125	>250	>250	>250	>250	
	**8c**	62.5	125	62.5	31.25	31.25	**0.49**	>500	>500	>500	>500	ND
		125	250	62.5	125	62.5	**0.49**	>500	>500	>500	>500	
	**8d**	32	125	62.5	>125	>125	>125	>125	>125	>125	>125	**13.8**
		62.5	125	125	>125	>125	>125	>125	>125	>125	>125	
	**8e**	32	62.5	62.5	15.62	**3.9**	125	15.62	31.25	**31.25**	62.5	20.4
		62.5	125	125	62.5	**7.81**	125	15.62	31.25	**31.25**	250	
	**8f**	62.5	125	62.5	31.25	**1.95**	125	>500	>500	>500	>500	**12.3**
		125	250	125	125	**3.9**	125	>500	>500	>500	>500	
	**8g**	62.5	32	62.5	31.25	**1.95**	125	>500	>500	>500	>500	ND
		62.5	32	62.5	125	**3.9**	125	>500	>500	>500	>500	
**GROUP 2**	**8h**	**8**	125	125	>125	125	>125	>500	>500	>500	>500	ND
		**16**	125	250	>125	125	>125	>500	>500	>500	>500	
	**8i**	32	62.5	62.5	>125	>125	>125	**7.81**	15.62	31.25	**31.25**	ND
		32	62.5	62.5	>125	>125	>125	**15.62**	15.62	250	**150**	
	**8j**	32	62.5	62.5	>125	62.5	62.5	**7.81**	**7.81**	31.25	250	ND
		32	62.5	62.5	>125	125	62.5	**7.81**	**7.81**	125	500	
	**8k**	**16**	**32**	32	125	125	125	>500	>500	>500	>500	29.8
		**16**	**32**	62.5	>125	>125	>125	>500	>500	>500	>500	
	**8l**	**16**	**32**	**32**	125	125	125	>125	>125	>125	>125	33.7
		**16**	**32**	**32**	>125	>125	125	>125	>125	>125	>125	
**GROUP 3**	**8m**	125	62.5	125	>125	>125	>125	>125	>125	>125	>125	ND
		500	500	500	>125	>125	>125	>125	>125	>125	>125	
	**8n**	32	32	62.5	>125	>125	>125	>500	>500	>500	>500	ND
		32	62.5	500	>125	>125	>125	>500	>500	>500	>500	
	**8o**	32	62.5	32	>125	>125	>125	>500	>500	>500	>500	ND
		32	125	62.5	>125	>125	>125	>500	>500	>500	>500	
	**8p**	16	62.5	32	>125	>125	>125	>500	>500	>500	>500	ND
		32	125	62.5	>125	>125	>125	>500	>500	>500	>500	
	**8q*^e^***	125	125	250	>125	>125	>125	>500	>500	>500	>500	74.2
		>1000	>1000	>1000	>125	>125	>125	>500	>500	>500	>500	
	**8r*^e^***	125	125	250	>125	>125	>125	>500	>500	>500	>500	ND
		>1000	>1000	>1000	>125	>125	>125	>500	>500	>500	>500	
**GROUP 4**	**8s**	62.5	62.5	62.5	>125	>125	>125	>125	>125	>125	>125	26.2
		62.5	62.5	125	>125	>125	>125	>125	>125	>125	>125	
	**8t**	62.5	62.5	62.5	>125	>125	>125	>125	>125	>125	>125	ND
		62.5	62.5	125	>125	>125	>125	>125	>125	>125	>125	
	**8u**	32	250	62.5	500	500	500	>125	>125	>125	>125	31.2
		32	250	125	>500	>500	>500	>125	>125	>125	>125	
	**8v**	32	250	**32**	500	500	500	**3.9**	**7.81**	62.5	62.5	ND
		32	250	**32**	>500	>500	>500	**15.62**	**15.62**	125	62.5	
**GROUP 5**	**9a**	62.5	125	125	>250	>250	>250	250	500	250	500	61.5
		62.5	125	125	>250	>250	>250	500	500	250	500	
	**9b**	32	125	125	250	250	250	**7.81**	**7.81**	**1.95**	500	ND
		62.5	125	125	250	250	250	**7.81**	**7.81**	**1.95**	500	
	**9c**	62.5	62.5	62.5	>125	>125	>125	>500	>500	>500	>500	23.8
		62.5	125	62.5	>125	>125	>125	>500	>500	>500	>500	
	**9d**	62.5	62.5	62.5	>125	>125	**7.81**	>500	>500	>500	>500	ND
		62.5	62.5	62.5	>125	>125	**7.81**	>500	>500	>500	>500	
	**9e**	32	32	32	125	125	125	>250	>250	>250	>500	ND
		32	62.5	62.5	125	125	125	>250	>250	>250	>500	
	**9f**	32	**32**	**32**	>125	>125	>125	>500	>500	>500	>500	23.5
		32	**32**	**32**	>125	>125	>125	>500	>500	>500	>500	
**GROUP 6**	**9g**	ND	ND	ND	ND	ND	ND	ND	ND	ND	ND	24
	**9h**	ND	ND	ND	ND	ND	ND	ND	ND	ND	ND	**14.2**
	**9i**	ND	ND	ND	ND	ND	ND	ND	ND	ND	ND	37.3
	**9j**	ND	ND	ND	ND	ND	ND	ND	ND	ND	ND	35.7
**INH**		0.5	>250	>250	–	–	–	–	–	–	–	–
		0.5	>250	>250								
**FLU**		–	–	–	0.12	3.91	1.95	–	–	–	–	–
					>125	15.62	3.91					
**PEN**		–	–	–	–	–	–	0.24	125	31.62	7.81	–
								0.24	125	125	15.62	
**CPF**		–	–	–	–	–	–	0.98	500	250	0.98	–
								0.98	500	250	0.98	
**DCMU**		–			–	–	–	–	–	–	–	1.9

The MIC determinations were performed according to the CLSI reference protocol: *^a^*mycobacteria (IC_90_ value), *^b^*M27-A2 for yeasts (IC_80_ value), *^c^*M38-A for moulds-fungi (IC_50_ value) and *^d^*bacteria (IC_90_ value), *^e^*compounds precipitated during the experiments.

Compounds **8h** and **8k**/**l** (Group 2) exhibited the highest activity against *M. tuberculosis*. Based on these observations, it can be concluded that chlorination in the C_(4)_ position results in less active compounds, substitution in the position C'_(4)_ of the anilide part is essential for high activity, and 4-Cl (Group 2) is more advantageous than 4-Br (Group 4). In general, compounds within Group 1 (3-Cl substituted) showed negligible activity. The influence of chlorine in the position C'_(4)_ of the anilide part seems to be dominant in Group 3 (series 5-Cl/3,4-Cl), see activity in Table 2. Lipophilicity seems to be only a secondary parameter; in the same way, the use of electronic parameter σ of R^2^ substituent does not improve the results of SAR discussion. High activity of **8h** cannot be explained on the basis of simple pattern.

Compounds **8k**/**l** and **8g** (Group 2 and Group 1) and **9e**/**f** (Group 5) showed the highest activity against M. avium and other compounds **8i**/**j**, **8a**, **8s** and **9d** demonstrated medium activity. Based on these facts, it can be assumed that the substitution of R^3^, especially by benzyl moiety, as well as preferential substitution by chlorine, especially in C'_(4)_ position of the anilide part of a molecule, is important for high activity. Chlorination of C_(4)_ or C_(5)_ position is only a secondary parameter. Substitution in C'_(4)_ by bromine (Group 4) or disubstitution in C'_(3,4)_ (Group 3) by chlorine causes activity decrease compared with Group 2. Lipophilicity seems to be also one of the parameters affecting antimycobacterial activity. The most active compounds from all groups show log k about 0.70. Stereoisomerism considerably influences antimycobacterial activity against M. avium as discussed below.

Compounds **8l**, **8v** and **9f** (Group 2, Group 4 and Group 5) showed the highest antimycobacterial activity against *M. kansasii*, and other compounds, **8k**, **9e**, **8i**/**j**, **8g** and **8c**, expressed substantial activity as well. Based on these facts, it can be concluded that chlorination of C_(4)_ or C_(5)_ position is only a secondary parameter, while substitution of C'_(4)_ position is more advantageous for high activity, and substitution by chlorine is preferred. Disubstitution of C'_(3,4)_ position is connected with activity decrease. The substitution of R^3^, particularly by benzyl or isopropyl moiety, is associated with higher activity, which can be positively influenced by lipophilicity increase. Similarly, based on Table 1 and Table 2 it can be considered that antimycobacterial activity against *M. kansasii* is also partly affected by bulk parameters expressed as MR (reflecting bulkiness) of R^3^ substituents [33]. It seems that antimycobacterial activity is increased by the increase of the bulkiness of individual R^3^ substituents in the molecule.

**Figure 2:**
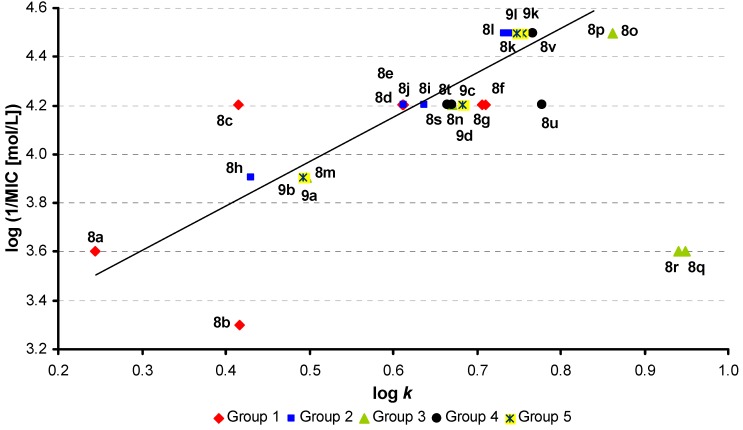
Dependence of antimycobacterial activity against *M. kansasii* (log 1/MIC [mol/L], 14 h experiment) on compounds lipophilicity expressed as log *k*.

From the set of 28 tested compounds the dependence of activity (log 1/MIC, 14 h experiment) on log *k* related to antimycobacterial activity against *M. kansasii* (see Figure 2) was linear for 24 compounds (compounds **8b**/**c** and **8q**/**r** were excluded from the correlation). Introduction of σ parameter did not improve the results of statistical analysis:

log(1/MIC) = 3.186(±0.091) + 1.574(±0.136) log *k*

r = 0.926, s = 0.092, F = 132.07, n = 24(1)

log (1/MIC) = 3.232(±0.095) +1.594(±0.135) log *k* – 0.185(±0.135) σ

r = 0.933, s = 0.090, F = 70.12, n = 24(2)

A very important parameter influencing activity is stereoisomerism, because individual enantiomers demonstrate considerable difference in their antituberculotic/antimycobacterial activity, e.g. enantiomers **8b**/**c**, **8f**/**g**, **8k**/**l**, **8o**/**p**, **8u**/**v**, **9c**/**d** and **9e**/**f** showed different antimycobacterial activity. These differences are especially evident for antimycobacterial activity against the atypical strains *M. avium* and *M. kansasii*. More often (*R*)-enantiomers showed higher activity. The determined differences in antimycobacterial activity for individual *R*/*S*-enantiomers cannot be explained on the basis of the results presented here. Due to these facts it can be assumed that stereospecific bond is formed between the compound and an enzyme in *Mycobacterium* sp. with subsequent enzyme inhibition.

#### 2.3.2. *In vitro* antifungal susceptibility testing

The evaluation of *in vitro* antifungal activity of the synthesized compounds was performed against eight fungal strains, but only activity against *Candida tropicalis* 156 (CT), *C. krusei* ATCC 6258 (CK) and against *Trichophyton mentagrophytes* 445 (TM) is shown in Table 2. Generally, all the compounds expressed only moderate antifungal activity, which was caused by low solubility of compounds in the testing medium and their precipitation during the incubation period, therefore no definite structure-activity relationships could be established. Only four compounds from Group 1, **8f**/**g**, **8e** and **8b**/**c**, showed very high antifungal activity comparable with or higher than the standard fluconazole (FLU) against *C. krusei* and against *T. mentagrophytes* 445. More often (*R*)-enantiomers showed higher activity, e.g. **8c**/**b**, **8e**/**d**, **9d**/**c**, nevertheless, the determined differences in antibacterial activity for individual *R*/*S*-enantiomers cannot be explained on the basis of the results presented here.

Generally, according to the results from Table 2, it can be concluded that chloration of C_(4)_ position or disubstitution R^2^ = 3,4-Cl considerably decreases solubility, and therefore it is not advantageous. The substitution R^2^ = 3-Cl is essential for high activity. The substitution of R^3^ modulates lipophilicity, and it seems to be an important parameter. Based on Table 2 it can be concluded that a lipophilicity decrease results in a decrease of antifungal activity: log *k* = 0.70 (**8f**/**g**) > 0.61 (**8e**) > 0.41 (**8b**/**c**).

When the dependence of activity (log 1/MIC, 24 h experiment) on log *k* related to antifungal activity of tested compounds against *C. krusei* (which is the most inhibited fungi strain) was studied, the dependence was linear only for Group 1 (3-Cl substituted derivatives):

log (1/MIC) = 2.901(±0.085) + 3.986(±0.149) log *k*

r = 0.998, s = 0.061, F = 711.38, n = 5(3)

The above discussed antimycobacterial activity against *M. kansasii* for the whole series of the tested compounds is mainly dependent on lipophilicity. In contrast to the antimycobacterial activity, the effect of σ parameter of R^2^ substituent on the activity of evaluated compounds against *C. krusei* was much more expressive than that of log *k*:

log (1/MIC) = 1.732(±0.780) + 8.863(±2.575) σ

r = 0.754, s = 0.614, F = 11.84, n = 11(4)

log (1/MIC) = 4.276 (±1.070) + 0.102 (±1.683) log *k*

r = 0.203, s = 0.934, F = 0.004, n = 11(5)

Multilinear correlation was found to fit the biological activity of compounds as a function of compound lipophilicity log *k* and σ constant of R^2^ substituent, and the results of statistical analysis showed that the activity was affected by both parameters.

log (1/MIC) = -0.038 (±1.185) + 1.986 (±1.077) log *k* + 10.743(±2.507) σ

r = 0.834, s = 0.546, F = 9.19, n = 11(6)

#### 2.3.3. *In vitro* antibacterial susceptibility testing

Twenty-eight benzamides were tested for their *in vitro* antibacterial activity against four Gram positive bacterial strains *Staphylococcus aureus* CCM 4516/08 (SA), methicilin resistant *S. aureus* H 5996/08 (MRSA), *S. epidermidis* H 6966/08 (SE) and *Enterococcus sp.* J 14365/08 (EF). The results are shown in Table 2. Generally, all the compounds expressed only moderate antibacterial activity caused by the low solubility of compounds in the testing medium and their precipitation during the incubation period, as it was mentioned above. In summary, five compounds **8v**, **8j**, **8e** and **9b** showed notable antibacterial activity comparable with or higher than the both standards penicillin G and ciprofloxacin against *S. aureus* methicilin resistant and *S. epidermidis*.

Based on the results from Table 2, especially halogenation of C'_(4)_ position (R^2^ = 4-Br or 4-Cl) seem to be important for antibacterial activity. Chloration of C_(4)_ position decreased solubility compared with chloration of C_(5)_ position. Stereoisomerism is probably also an important parameter influencing the activity, because individual enantiomers demonstrate considerable difference in their antibacterial activity, e.g., enantiomers **8u**/**v**, **8d**/**e**, **8i**/**j** and **9a**/**b**. More often (*R*)-enantiomers showed higher activity (the same as *M. kansasii*). R^3^ substitution modulates physico-chemical properties of the discussed compounds. According to Table 2, a bulkier substituent is probably more advantageous. A more detailed SAR study cannot be established due to the small number of effective compounds.

#### 2.3.4. Inhibition of photosynthetic electron transport (PET) in spinach chloroplasts

Seventeen compounds were tested for their PET in spinach (*Spinacia oleracea* L.) chloroplasts, and they showed some wide-range activity, see Table 2. Compounds **8f** (IC_50_ = 12.3 µmol/L), **8e** (IC_50_ = 13.8 µmol/L) and **9h** (IC_50_ = 14.2 µmol/L) were the most effective inhibitors of the tested compounds, which PET-inhibiting activity in spinach chloroplasts was measured. The activity of the rest of the studied compounds was moderate or low relative to the standard. 

Due to the fact that PET-inhibiting data are available only from Group 1 and Groups 5 + 6, SAR study involves the first of these series, but a general SAR hypothesis may be proposed. Generally, it can be concluded that C_(4)_ substitution is more advantageous then C_(5)_ substitution. In the C_(5)_ series C´_(3)_ substitution is important for PET activity. Compounds within the C_(5)_ series with substitution in C´_(4)_ by Cl or Br exhibit similar activity, which is lower than that of C´_(3)_ substituted compounds, but solubility in the testing medium is drastically reduced in the case of C´_(4)_ substitution. Disubstitution in C´_(3,4)_ by Cl also causes lower solubility. Bulkiness and branching of R^3^ substitution are also important. It can be noticed that mainly (*S*)-enantiomers are active in PET assay (compare **8d**/**e** or **8k**/**l**) and only **8e**, **9f**, **9g** are slightly active (*R*)-enantiomers. The correlations between PET-inhibiting activity and lipophilicity could be expressed by equation:

log (1/IC_50_) = 1.925(±0.427) + 8.302(±1.497) log *k* – 6.270(±1.266) (log *k*)^2^

r = 0.856, s = 0.191, F = 19.17, n =17(7)

For PET inhibition of tested compounds the lipophilicity was determinant because the introduction of σ parameter did not improve the results of statistical analysis:

log (1/IC_50_) = 1.903(±0.460) + 8.365(±1.595) log *k* – 6.345(±1.383) (log *k*)^2^ + 0.0476(±0.279) σ

r = 0.856, s = 0.198, F = 11.91, n = 17(8)

## 3. Experimental

### 3.1. Synthesis

The studied compounds are shown in Scheme 1 and listed in Table 1. Synthesis and characterization of the discussed chloro-2-hydroxy-*N*-[2-(arylamino)-1-alkyl-2-oxoethyl]benzamides were described in reference [14].

### 3.2. Lipophilicity determination by HPLC (capacity factor k/calculated log k)

A Waters Alliance 2695 XE HPLC separation module and a Waters Photodiode Array Detector 2996 (Waters Corp., Milford, MA, USA) were used. A Symmetry^®^ C_18_ 5 μm, 4.6 × 250 mm, Part No. WAT054275 (Waters Corp., Milford, MA, USA) chromatographic column was used. The HPLC separation process was monitored by Empower™ 2 Chromatography Data Software, Waters 2009 (Waters Corp., Milford, MA, USA). A mixture of MeOH p.a. (70%) and H_2_O-HPLC – Mili-Q Grade (30%) was used as a mobile phase. The total flow of the column was 1.0 mL/min, injection volume, 30 μL, column temperature, 30 °C and sample temperature, 10 °C. The detection wavelength of 210 nm was chosen. The KI methanolic solution was used for the dead time (t_D_) determination. Retention times (t_R_) were measured in minutes. The capacity factors *k* were calculated using the Empower™ 2 Chromatography Data Software according to formula *k* = (t_R_ - t_D_)/t_D_, where t_R_ is the retention time of the solute, whereas t_D_ denotes the dead time obtained using an unretained analyte. Log *k*, calculated from the capacity factor *k*, is used as the lipophilicity index converted to log *P* scale. The log *k* values of the individual compounds are shown in Table 1.

### 3.3. Lipophilicity calculations

Log *P*, *i.e.* the logarithm of the partition coefficient for *n-*octanol/water, was calculated using the programs CS ChemOffice Ultra ver. 10.0 (CambridgeSoft, Cambridge, MA, USA) and ACD/LogP ver. 1.0 (Advanced Chemistry Development Inc., Toronto, Canada). Clog *P* values (the logarithm of *n*-octanol/water partition coefficient based on established chemical interactions) were generated by means of CS ChemOffice Ultra ver. 10.0 (CambridgeSoft, Cambridge, MA, USA) software. The results are shown in Table 1.

### 3.4. In vitro antimycobacterial evaluation

The *in vitro* antimycobacterial activity of all prepared compounds was evaluated against *Mycobacterium tuberculosis* MTB CNCTC My 331/88 (identical with H37RV and ATCC 27294, dilution of the strain was 10^−3^ μmol/L), *Mycobacterium avium* MA CNCTC My 330/88 (identical with ATCC 25291, dilution of the strain was 10^−5^ μmol/L) and *Mycobacteriumkansasii* MK CNCTC My 235/80 (identical with ATCC 12478, dilution of the strain was 10^−4^ μmol/L) in the Laboratory for mycobacterial diagnostics and TB, Institute of Public Health in Ostrava, Czech Republic. All strains were obtained from the Czech National Collection of Type Cultures (CNCTC). Antimycobacterial activities were determined in the Sula semisynthetic medium (Sevac, Prague, Czech Republic). Each strain was simultaneously inoculated into a Petri plates containing the Lowenstein-Jensen medium for the control of sterility of the inoculum and its growth. The tested compounds were added to the medium as DMSO solutions. The following concentrations were used: 250, 125, 62, 32, 16, 8, 4, 2, and 1 μmol/L. Inoculated plates kept in microtone bags were incubated at 37 °C. Reading was carried out on a stand with a bottom magnifying mirror, macroscopically, with the use of magnifying glass. The growth in plates was evaluated after 14 and 21 days of the incubation. The growth of the colonies in a control well plate, corresponding to the growth into 100 colonies in the control Lowenstein-Jensen medium was considered as an optimum dilution for the evaluation of the results. In the course of the test evaluation, the MIC (μmol/L) definition was considered as the lowest substance concentration at which the inhibition of growth of mycobacteria occurred. [34] Isoniazid (INH) was used as the standard as it is a clinically used anti-mycobacterial drug. The results are shown in Table 2. The MIC for mycobacteria was defined as a 90% or greater (IC_90_) reduction of growth in comparison with the control. The MIC/IC_90_ value is routinely and widely used in bacterial assays and is a standard detection limit according to the Clinical and Laboratory Standards Institute (CLSI, formerly NCCLS, www.clsi.org/).

### 3.5. In vitro antifungal susceptibility testing

The broth microdilution test [35,36,37] was used for the assessment of *in vitro* antifungal activity of the synthesized compounds against *Candida albicans* ATCC 44859, *C. tropicalis* 156, *C. krusei* E28, *C. glabrata* 20/I, *Trichosporon asahii* 1188, *Trichophyton mentagrophytes* 445, *Aspergillus fumigantus* 231 and *Absidia corymbifera* 272. Fluconazole (FLU) was used as a standard, since it is a clinically used antimycotic drug. The procedure was performed with a two-fold dilution of the compounds in RPMI 1640 (Sevapharma a.s., Prague, Czech Republic) buffered to pH 7.0 with 0.165 mol of 3-morpholino-propane-1-sulfonic acid (MOPS, Sigma, Germany). The final concentrations of the compounds ranged from 500 to 0.975 μmol. Drug–free controls were included. The MIC determination was performed according to the CLSI reference protocol M27-A2 for yeasts (IC_80_ value) and M38-A for moulds (IC_50_ value). IC_50_ values were defined as a 50% reduction of growth in comparison with the control, and IC_80_ values were defined as a 80% reduction of growth in comparison with the control. The trays were incubated at 35 °C, and MICs were read visually for filamentous fungi and photometrically for yeasts as absorbance at 540 nm after 24 h and 48 h. The MIC values for the dermatophytic strain (*T. mentagrophytes*) were determined after 72 h and 120 h. MICs were determined twice and in duplicate. The results are summarized in Table 2.

### 3.6. In vitro antibacterial susceptibility testing

The synthesized compounds were evaluated for *in vitro* antibacterial activity against *Staphylococcus aureus* CCM 4516/08, methicilin resistant *Staphylococcus aureus* H 5996/08, *Staphylococcus epidermidis* H 6966/08 and *Enterococcus sp.* J 14365/08. Penicillin G (PEN) and ciprofloxacin (CPF) were used as standards since they are clinically used antibacterial drugs. All strains were sub-cultured on nutrient agar (HiMedia) and maintained on the same medium at 4 °C. Prior to testing, each strain was passaged onto nutrient agar and bacterial inocula were prepared by suspending a small portion of bacterial colony in sterile 0.85% saline. The cell density was adjusted to 0.5 McFarland units using a densitometer (Densi-La-Meter, PLIVA Lachema Diagnostika, Czech Republic). The final inoculum was made by 1:20 dilution of the suspension with the test medium (Mueller-Hinton broth). The compounds were dissolved in DMSO and the anti-bacterial activity was determined using Mueller-Hinton broth (MH broth, HiMedia, pH 7.0 ± 0.2). Controls consisted of MH broth and DMSO alone. The final concentration of DMSO in the MH broth did not exceed 1% (v/v) of the total solution composition. The activity of the studied compounds was determined as the minimal inhibition concentration (MIC) according to NCCLS guidelines [38] using broth microdilution test. The MICs were defined as 90% inhibition of bacterial growth compared to the control and were determined after 24 and 48 h of static incubation at 37 °C. After incubation MICs were read visually as an absorbance at 540 nm. The results are shown in Table 2.

### 3.7. Study of inhibition photosynthetic electron transport (PET) in spinach chloroplasts

Chloroplasts were prepared from spinach (*Spinacia oleracea* L.) according to Masarovicova and Kralova [39]. The inhibition of photosynthetic electron transport (PET) in spinach chloroplasts was determined spectrophotometrically (Genesys 6, Thermo Scientific, USA), using an artificial electron acceptor 2,6-dichlorophenol-indophenol (DCIPP) according to Kralova *et al.* [40], and the rate of photosynthetic electron transport was monitored as a photoreduction of DCPIP. The measurements were carried out in phosphate buffer (0.02 mol/L, pH 7.2) containing sucrose (0.4 mol/L), MgCl_2_ (0.005 mol/L) and NaCl (0.015 mol/L). The chlorophyll content was 30 mg/L in these experiments and the samples were irradiated (~100 W/m^2^) from 10 cm distance with a halogen lamp (250 W) using a 4 cm water filter to prevent warming of the samples. The studied compounds were dissolved in DMSO due to their limited water solubility. The applied DMSO concentration (up to 4%) did not affect the photochemical activity in spinach chloroplasts. The inhibitory efficiency of the studied compounds was expressed by IC_50_ values, *i.e.* by molar concentration of the compounds causing 50% decrease in the oxygen evolution rate relative to the untreated control. The comparable IC_50_ value for a selective herbicide 3-(3,4-dichlorophenyl)-1,1-dimethylurea, DCMU (Diurone^®^) was about 1.9 μmol/L [41]. The results are summarized in Table 2.

## 4. Conclusions

A series of twenty-two 5-chloro-2-hydroxy-*N*-[2-(arylamino)-1-alkyl-2-oxoethyl]benzamides and ten 4-chloro-2-hydroxy-*N*-[2-(arylamino)-1-alkyl-2-oxoethyl]benzamides is described. Their lipophilicity was determined using a well established RP-HPLC method. The prepared compounds were tested for their antimycobacterial, antifungal and antibacterial activity as well as for their ability to inhibit photosynthetic electron transport (PET) in spinach chloroplasts (*Spinacia oleracea* L.). For all the compounds the relationships between their lipophilicity and chemical structure as well as structure-activity relationships were discussed. *N*-{(1*R*)-1-benzyl-2-[(4-chlorophenyl)amino]-2-oxo-ethyl}-5-chloro-2-hydroxybenzamide (**8l**, 32 μmol/L) and *N*-{(1*R*)-1-benzyl-2-[(4-chlorophenyl)-amino]-2-oxoethyl}-4-chloro-2-hydroxybenzamide (**9f**, 32 μmol/L) showed the highest antitubercular activity and higher antimycobacterial effect than the standard isoniazid. *N*-{(1*S*/*R*)-1-benzyl-2-[(3-chlorophenyl)amino]-2-oxoethyl}-5-chloro-2-hydroxybenzamide (**8f**, **8g**, 3.9 μmol/L) and 5-chloro-*N*-[(1*R*)-1-{[(3-chlorophenyl)amino]-carbonyl}-2-methylpropyl)]-2-hydroxybenzamide (**8e**, 7.81 μmol/L) demonstrated higher antifungal activity against *Candida krusei* and 5-chloro-*N*-{(1*R*)-2-[(3-chlorophenyl)amino]-1-methyl-2-oxoethyl}-2-hydroxybenzamide (**8c**, 0.49 μmol/L) showed higher antifungal activity against *Trichophyton mentagrophytes* than the standard fluconazole. 5-Chloro-*N*-[(1*S*/*R*)-1-{[(4-chlorophenyl)amino]carbonyl}-2-methylpropyl)]-2-hydroxybenzamide (compound **8j**, 7.81 μmol/L) exhibited higher activity against methicillin-resistant *Staphylococcus aureus* than penicillin G and ciprofloxacin. 4-Chloro-*N*-{(1*R*)-2-[(4-chlorophenyl)-amino]-1-methyl-2-oxoethyl}-2-hydroxy benzamide (**9b**, 7.81 μmol/L and 1.95 μmol/L) showed higher activity against methicillin-resistant *Staphylococcus aureus* and *S. epidermidis* than penicillin G and ciprofloxacin. *N*-{(1*S*)-1-benzyl-2-[(3-chlorophenyl)amino]-2-oxoethyl}-5-chloro-2-hydroxybenz-amide (**8f**, 12.3 μmol/L) and 5-chloro-*N*-[(1*S*)-1-{[(3-chlorophenyl)amino]-carbonyl}-2-methylpropyl)]-2-hydroxybenzamide (**8d**, 13.8 μmol/L) showed the highest PET-inhibition activity among the discussed compounds. It can be concluded that (*R*)-enantiomers demonstrated higher antimicrobial activity compared with (*S*)-enantiomers, while (*S*)-enantiomers showed higher inhibition of photosynthetic electron transport in spinach chloroplasts.
